# Element concentration, stable and radioactive isotope data from multiple environmental matrices in two mid-sized Central-European river basins

**DOI:** 10.1016/j.dib.2026.112955

**Published:** 2026-06-11

**Authors:** Máté Krisztián Kardos, Adrienne Clement, Zsolt Jolánkai, Ildikó Musa, Radmila Milačič Ščančar, Janez Ščančar, Miroslav Štrbac, Katarina Kozlica, Polona Vreča, Klara Žagar, Sonja Lojen, József Deák, Palcsu Laszlo, Elemér László, István Gábor Hatvani, Zoltán Kern

**Affiliations:** a Budapest University of Technology and Economics, Faculty of Civil Engineering, Department of Sanitary and Environmental Engineering Műegyetem rkp. 3., H-1111 Budapest, Hungary; b Department of Environmental Sciences, Jožef Stefan Institute. Jamova cesta 39, SI-1000 Ljubljana, Slovenia; c GWIS Ltd: GWIS Ltd for Environmental Protection and Groundwater Quality. Hóvirág u. 9., H-8200 Veszprém, Hungary; d ATOMKI: HUN-REN Institute for Nuclear Research. Bem tér 18/c, H-4026 Debrecen, Hungary; e IGGR: HUN-REN RCAES Institute for Geological and Geochemical Research HUN-REN Research Centre for Astronomy and Earth Sciences. Budaörsi út 45., H-1112 Budapest, Hungary

**Keywords:** Water quality monitoring, Water stable isotopes, River basin monitoring, Potentially toxic elements, Rare earth elements, Fingerprinting

## Abstract

This article presents a comprehensive multi-matrix dataset collected to support the investigation and validation of catchment-scale contaminant transport processes using combined hydrochemical, multi-element, and isotopic approaches. The dataset was generated within a targeted monitoring program conducted in two medium-sized Central European catchments, the Koppány River (Hungary) and the Ledava River (Slovenia), both characterized by intensive agricultural land use, wastewater inputs, and erosion-sensitive conditions.

The dataset integrates measurements from multiple environmental compartments, including precipitation, surface water, groundwater, wastewater, soil, riverbed sediment, and suspended particulate matter (SS). Sampling was designed to capture both baseflow conditions and hydrologically dynamic periods, with particular emphasis on high-flow events associated with enhanced erosion and material transport. River water samples were collected through a combination of grab sampling and flow-proportional automatic sampling, while suspended sediments were obtained using passive samplers during flood events. Soil and sediment samples were collected at representative locations and processed to fine fractions prior to analysis.

The analytical data include conventional hydrochemical parameters (e.g. major ions, nutrients, pH, electrical conductivity, turbidity), alongside detailed multi-element composition comprising potentially toxic elements, rare earth elements, and geological normalizers. Elemental concentrations were determined using inductively coupled plasma mass spectrometry (ICP-MS) following microwave-assisted digestion or selective extraction procedures. In addition, isotope-based tracers were incorporated, including water stable isotopes (δ²H, δ¹⁸O), radioisotope data (e.g. ^3^H, ^14^C) indicating groundwater age information, and nitrate stable isotopes (δ¹⁵N_NO3_, δ¹⁵O_NO3_), enabling the characterization of hydrological pathways and nutrient sources.

The dataset combines newly acquired measurements with pre-existing hydrological, hydrochemical, and land use data into a harmonized database structure. This integration enables the comparison of elemental and isotopic signatures across environmental matrices and hydrological conditions, supporting applications such as source characterization, assessment of particulate and dissolved transport processes, and evaluation of hydrological flow components.

The data are provided with detailed metadata on sampling conditions, analytical procedures, and quality control, including the use of certified reference materials and replicate analyses. Limitations related to incomplete parameter coverage, variable hydrological data availability, and missing metadata for a subset of groundwater samples are documented.

This dataset provides a consistent, multi-tracer observational basis for the analysis of contaminant transport processes in agriculturally impacted catchments and for the evaluation of catchment-scale modelling approaches.

Specifications Table

**Table:** 

Subject	Earth & Environmental Sciences
Specific subject area	Hydrology / water quality: Monitoring of water cycle and contaminant transport processes on the catchment scale by analysing environmental samples
Type of data	Tables on elemental composition of precipitation, river water, wastewater, groundwater, as well as riverbed sediment, river suspended sediment and soil samples taken in the two pilot regions.
	Tables on waters stable isotope, nitrate stable isotope as well as tritium and radiocarbon concentration of precipitation, river water, wastewater and groundwater samples. Raw concentration and content analysis results
Data collection	In both catchments, following types of samples were collected. (i) precipitation samples (monthly composites); (ii) river water samples (total and dissolved concentrations under low-flow and high-flow separately); (iii) groundwater samples from both shallow and deep wells; (iv) riverbed sediment samples; (v) river suspended sediment (vi) soil samples and (vii) wastewater effluents. Apart of basic chemistry (e.g. ion composition), samples were analysed for a list of 49 elements with ICP-MS. In addition, water and nitrate stable isotopic composition was determined by isotope ratio mass spectrometry. Selected samples were analysed for tritium activities using ^3^He-ingrowth method and radiocarbon activities of dissolved inorganic carbon using accelerator mass spectrometry.
Data source location	Environmental samples were collected in two mid-size Central European River catchments: the Koppány River Basin upstream to Tamasi, Somogy county, SW-Hungary (area: 662 km²) and the 895 km² Ledava River Basin located around Murska Sobota, at the Hungarian-Slovenian country border. Coordinates for centroids of the river basins are 18.066E 46.635 N and 16.261E 46.692 N, respectively. Bounding box coordinates are 17.793E, 46.494 N, 18.308E, 46.768 N for the Koppany and 15.932E, 46.479 N 16.571E, 46.904 N for the Ledava catchment.
Data accessibility	Repository name: HUN-REN Data Repository Platform DOI: 10.5158/ARP/4LHO5O Direct URL to data: https://repo.researchdata.hu/file.xhtml?persistentId=hdl:21.15109/ARP/4LHO5O/LYL8WX&version=1.0 [1].
Related research article	Radmila Milačič Ščančar, Janez Ščančar, Katarina Kozlica, Miroslav Štrbac, Mavro Lučić, Zsolt Jolánkai, Máté Krisztián Kardos, Adrienne Clement: Spatial distribution and catchment-scale controls of potentially toxic elements, phosphorus and rare earth elements in the Koppány (Hungary) and Ledava (Slovenia) rivers [*under 2nd round of review in: Environmental Sciences Europe*]

## 1 Value of the Data

• Data provide information on the chemical composition of environmental media for the studied catchments in a so far unprecedented detail.
• It might encourage researchers to look for hidden relationships between environmental matrixes / elements through multivariable analysis methods and beyond.
• Data provide the basis for a comparative isotope hydrological study on surface, shallow and deep groundwater, supporting science-based water management planning of the surveyed Hungarian and Slovenian lowland catchments.
• Isotope in precipitation data will be used for the update existing three-year dataset for the Murska Sobota available at the SLONIP platform [https://slonip.ijs.si/] and represent an important input function for further water cycle investigations (estimation of recharge area, mean residence times, climate change response, etc.). Together with neighboring data, the reported water isotope data will enable the creation of more reliable regional and global isoscapes for precipitation and groundwater.
• Nitrate isotope data can be used to attribute river nitrate loading to organic and inorganic sources.

## 2 Background

The dataset was compiled within a research project focused on improving the validation of catchment-scale contaminant transport models by integrating detailed field observations with tracer-based approaches. The motivation arises from commonly used catchment emission models, which provide limited information on contaminant sources, pathways, and temporal dynamics [2].

The methodological background combines two established approaches. Multi-element geochemical fingerprinting uses the composition of potentially toxic elements (PTE), rare earth elements (REE), and geological normalizers (GN) to distinguish between sources and to trace sediment-associated transport processes [[3], [4], [5]]. Environmental isotope techniques complement this by characterizing water origin and movement: water stable isotopes (δ²H, δ¹⁸O) support runoff component separation [6], ³H and ^14^C provide groundwater residence time, and nitrate isotopes (δ¹⁵N_NO3_ and δ¹⁵O_NO3_) help differentiate nitrogen sources and transformations [7,8].

The dataset integrates measurement results across multiple environmental compartments (precipitation, surface water, groundwater, soil, sediment, and suspended solids). Sampling was designed to capture various river flow conditions. Monitoring data were combined with ancillary information on hydrometeorology, land use, and anthropogenic inputs to support consistent statistical analysis and comparison with model frameworks.

## 3 Data Description

**Fig. 1:**
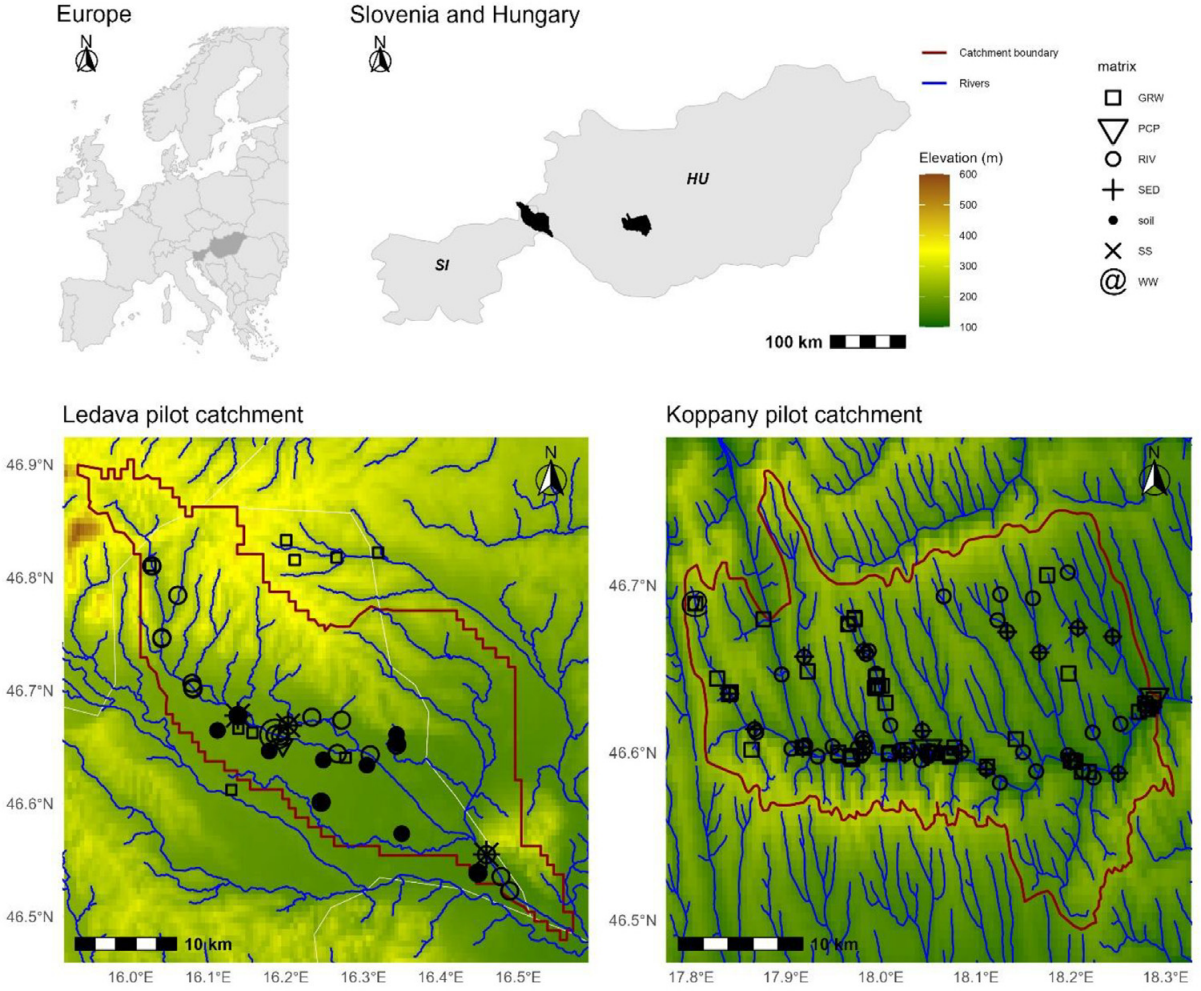
Sampling locations in the Ledava (bottom left) and Koppany (bottom right) catchments. Exact locations for the unique media are visualized, although in some cases overlapping. Inset maps in the upper panel show the study area in Europe and the catchments within Slovenia and Hungary.

The database contains data for 2 and 1 precipitation (PCP), 55 and 10 river (RIV), 93 and 8 groundwater (GRW), and 1–1 wastewater (WW) sampling station(s), in the Koppány and Ledava catchments, respectively (Fig. 1). Sampling campaigns were carried out for river water between 12.07.2022 and 21.07.2025, while for groundwater between 18.04.2023 and 27.10.2025. Precipitation was sampled on a monthly basis.

All data on sampling campaigns, locations, sampled media, and the results of sample analysis are compiled in a single MS Excel table. The 1st sheet is a title page (“General description”); followed by two types of spreadsheets: (i) those containing describing (meta) data and (ii) the sheets containing the measurement results themselves.

To ensure long-term usability and facilitate data exchange among researchers, the metadata structure has been standardized across worksheets with measurement results. Sampling locations are assigned unique identifiers (“locationID”) and geographical coordinates in the WGS system. To each sample, a unique sample identifier is assigned, sampling date (start and end in case of PCP) as well as further, matrix-specific columns. Analytical methods for each determinand and laboratory are described on the “Measurements” sheet. Parameter names and measurement units follow consistent conventions throughout the database. The structure follows a machine-readable format with clearly defined column headers, controlled vocabularies for sampling media, parameters and station categories, and standardized date and coordinate formats (ISO 8601 and WGS84, respectively). These measures facilitate integration with external databases, geographic information systems (GIS), or other research infrastructures. Table 1 lists the spreadsheets along with a short description of their contents.

**Table 1:** Name and content of sheets in the excel table.

Sheet name	Sheet content	number of records (without header)
General description	Brief general description of the dataset, including the sampling areas. Glossary Version number	NA
Measurements	Parameter abbreviations and parameter full names, measurement units, corresponding LOD and LOQ values, analysis methods, ISO norms (if applicable) across the various environmental media and the various laboratories	120
Campaigns	One-sentence description of the sampling campaigns: date, goal, number of locations visited / number of samples taken by environmental media	37
Locations	Sampling locations by environmental media: code (ID) of location, name (description) of location: town / river, geographical coordinates, further descriptor of location (e.g. well and groundwater depth in case of groundwater samples)	196
PCP	Precipitation samples and their measurement results – location ID, date of sampling, basic chemistry measurement results and water stable isotope composition of the samples	62
RIV	River water samples and their measurement results – location ID, sampling date, type of sample (grab / auto) flow situation (low flow / high flow), on-site observation records, basic chemistry analysis results, water stable isotopic composition, nitrate stable isotopic composition, radiocarbon and tritium measurement results, total and dissolved element measurement results	248
GRW	Groundwater samples and their measurement results – location ID, date of sampling, on-site observation records, basic chemistry analysis results, water stable isotope composition, nitrate stable isotopic composition, tritium and radiocarbon measurement results, dissolved element concentrations	152
WW	Wastewater samples and their measurement results. On-site observations, basic chemistry analysis results, water stable isotope composition, nitrate stable isotope composition, and total element concentrations.	5
soil_SS_SED	Riverbed sediment, suspended sediment and soil samples and their measurement results – matrix, sampling date, sample type, physico-chemical properties of the sample, radiocarbon isotope measurement results, nitrate stable isotope measurement results. Soil, sediment and SS element contents were determined for total concentrations, while soils and sediments were analysed also for fraction extractable in 0.11 mol/L acetic acid, to determine the easily soluble, mobile elemental fractions.	43

The database design explicitly follows the FAIR (Findable, Accessible, Interoperable, and Reusable) data principles. Findability is supported through comprehensive metadata descriptions, persistent station identifiers, and clear dataset documentation. Accessibility is ensured by providing human-readable documentation on the first sheet together with machine-readable data tables. Interoperability is achieved through the use of standardized metadata fields, controlled vocabularies, internationally recognized coordinate and date standards, and open exchange formats. Reusability is promoted by detailed methodological descriptions, traceable links between samples and analytical results, standardized units, quality-assurance information, and comprehensive metadata.

## 4 Experimental Design, Materials and Methods

### 4.1 Study design and sample collection

The experimental design followed a multi-compartment and multi-tracer approach, integrating hydrological, chemical, and geochemical measurements across seven environmental matrices: precipitation (PCP), river water (RIV), groundwater (GRW) and wastewater (WW), as well as soil, river suspended solids (SS) and riverbed sediment (SED).

Regarding PCP, samples were collected as a monthly composite. For GRW, four types of wells were sampled using the grab sampling method: existing shallow groundwater wells, existing deep groundwater wells, as well as freshly drilled holes either on agricultural fields close to the river or holes drilled in proximity of the river in the riverbed sediment (hyporheic zone sampling). Regarding WW, effluents from two major treatment plants (one in each pilot region) were sampled either by taking grab samples, or by taking 24-h time-proportional samples using a WaterSam WS Porti 2 autosampler.

River sampling (RIV) was structured to capture hydrological variability. Samples were collected under both low-flow (baseflow) and high-flow conditions. Low-flow sampling was performed by grab sampling, while samples representing high-flow conditions were taken on an event driven base, using automatic flow-proportional samplers in HU and grab samples in SI. Samples were collected in pre-cleaned polyethylene bottles. For dissolved element analysis, samples were filtered (0.45 µm) and acidified (HNO₃, pH <2) on site. For total concentrations, unfiltered samples were acidified in the same manner. All samples were stored frozen (−20 °C) until analysis. Continuous hydrological and water quality data (e.g. discharge, electrical conductivity) were obtained from automatic sensors to support the interpretation of the discrete samples.

Soil sampling locations in the Ledava catchment were selected to represent the main land use classes; samples were collected from nine representative locations (agricultural and forest land uses). At each site, at least five subsamples from the upper 30 cm were composited and reduced by quartering. In the Koppany catchment, soil samples were collected in frames of a previous project and have already been published [9] and are thus not discussed herein nor included in the referred database [1].

SS samples in HU were collected both during low-flow periods and during flood events, using Phillips-type passive samplers [10]. In SI, SS samples were obtained during high-flow events either by passive samplers, followed by freeze-drying, or by filtration (0.45 µm) of large water volumes (≈10 L), using vacuum pump and drying of samples at 40 °C. Riverbed samples were wet sieved (<1 mm, then <63 µm), and freeze-dried, or dried at 40 °C. The proportion of the <63 µm fraction was determined gravimetrically.

SED sampling locations were aligned with river monitoring sites to enable cross-matrix comparison. Riverbed sediments were collected by grab sampling the top 10 cm layer.

### 4.2 Measured parameters

The analytical program included (i) on-site parameters (“OS”: pH, dissolved oxygen, electrical conductivity, turbidity); (ii) basic chemistry (“BC”) like indicators of the organic household (chemical oxygen demand), major ions (carbonate forms, chloride, calcium, magnesium), and nutrients (nitrogen and phosphorus forms); (iii) “all” elements: (“AE”: 49 elements including potentially toxic elements, rare earth elements and geological normalizers); (iv) water stable isotopes (WSI, δ^2^H and δ^18^O); (v) tritium (^3^H) activity; (vi) carbon isotopes (“CI”: *δ*^13^C_DIC_ and ^14^C); and (vii) nitrate stable isotopes (NSI, δ ^15^N_NO3_ and δ ^18^O_NO3_). Table 2. summarizes the correspondence between environmental matrices and measured parameter groups and provides insight into the purpose of the different measurements.

**Table 2:** Description of the measurements executed in the two pilot areas.

Sampled matrix Goal of the measurement	Precipitation	River water	Ground water	Waste water	Soil, river suspended solids, and riverbed sediment
Determine the recharge from deeper aquifers during base-flow conditions		OS, BC, AE, WSI, CI	OS, BC, AE, WSI, T, CI		
Determine groundwater residence times and N legacy		BC, NSI	OS, BC, NSI		AE, CI, NSI
Separate surface water runoff and groundwater flow (base flow) in stream waters	BC, WSI	OS, BC, WSI	BC, WSI	OS, BC, AE, WSI, NSI	
Investigate the sources and transport pathways of contaminants	BC	OS, AE	BC, AE	AE	AE, NSI
Describe sediment and contaminant transport processes during runoff / high-flow events		AE, WSI	OS, BC, AE, WSI	AE, WSI, NSI	AE, NSI

OS = on-site parameters (pH, dissolved oxygen, electric conductivity). BC =basic chemistry: most common anions, cations and nutrient household indicators. AE = “all elements”, more precisely 49 elements of the periodic table, including potentially toxic elements; rare earth elements and geological normalizers. WSI = water stable isotopes (δ ^2^H, δ ^18^O). T = tritium (^3^H). CI = carbon isotopes (stable and radioactive). NSI = nitrate stable isotopes (δ ^15^N_NO3_ and δ ^18^O_NO3_).

### 4.3 Measurement of on-site parameters (“OS”)

On-site parameters were measured by the lab conducting the sampling with standard commercial products. BME has measured pH and electrical conductivity with WTW 3320, Xylem Analytics GmbH. Weilheim, Germany, and dissolved oxygen concentration with HACH HQ 1130, Hach Co., Loveland, Colorado, USA. JSI has determined on-site parameters using a 6PFCE Ultrameter II, Myron L Company, Carlsbad, CA, USA.

### 4.4 Measurement of basic chemistry (“BC”) parameters at BME and JSI

Immediately after sampling (practically on the next day), the analysis of the basic ionic composition as well as the nutrient concentration of the sample was conducted in local labs according to international standards described on the sheet “Measurements” of [1].

### 4.5 Measurement of total and dissolved element concentrations (“AE”) at JSI

For aqueous samples, total PTE concentrations were determined after microwave digestion, while dissolved concentrations were measured directly in filtered and acidified samples. All measurements were performed in triplicate. After being transported to the lab, soil samples were dried (40 °C) and sieved (<2 mm, then <150 µm).

Elemental concentrations were determined using an Agilent 7700x ICP-MS system. Sample digestion was performed with a CEM MARS 6 microwave digestion system. Sample weighing was performed with a Mettler AE 163 analytical balance, shaking using a Vibromix 40 orbital shaker, and centrifugation with a Hettich Universal 320 centrifuge.

Ultrapure water (18.2 MΩ·cm) and high-purity reagents (HNO₃, HCl, HF, H₂O₂, H₃BO₃, CH₃COOH) were used throughout. Calibration was performed using multi-element standard solutions and internal standards. Certified reference materials (SPS-SW1 and CRM 320R) were used for quality control, confirming analytical accuracy within ±3%.

Total elemental concentrations in soil, sediment, and SS were determined after microwave-assisted acid digestion (HNO₃–HCl–HF–H₂O₂ mixture), followed by boric acid treatment and dilution prior to ICP-MS analysis. Acetic acid-extractable fractions of potentially toxic elements (PTEs) were obtained by shaking 0.5 g of sample with 0.11 mol/L acetic acid for 16 h, followed by centrifugation, filtration, and ICP-MS measurement.

### 4.6 Measurement of water stable isotope (WSI) composition of the samples at JSI and IGGR

The hydrogen and oxygen isotopic compositions (*δ*^2^H and *δ*^18^O) were determined dominantly at the JSI (at the Jožef Stefan Institute) using a dual inlet isotope ratio mass spectrometer (DI IRMS, Finnigan MAT DELTA^plus^, Finnigan MAT GmbH, Bremen, Germany) with an automated H_2__—_H_2_O [11] and CO_2__—_H_2_O [12,13] equilibrator HDOeq48 Equilibration Unit (custom built by M. Jaklitsch, Vienna, Austria). All samples were measured in duplicate together with laboratory reference materials (LRMs) that were periodically calibrated against primary IAEA calibration standards VSMOW2 and SLAP2 to the VSMOW/SLAP scale. The results were normalized to the VSMOW/SLAP scale using the LIMS (Laboratory Information Management System for Light Stable Isotopes) programme and are expressed in the *δ* notation (‰) as mean and standard deviation [14,15]. For independent quality control, in-house LRMs with measurement uncertainties estimated using the Kragten method [16] and USGS commercial reference materials (USGS 45, USGS47) were used. The overall uncertainties were below 1‰ and 0.05‰ for *δ*^2^H and *δ*^18^O, respectively, and the average sample repeatability was 0.3‰ for *δ*^2^H and 0.02‰ for *δ*^18^O. The *δ*^2^H and *δ*^18^O values for a smaller set of precipitation and stream water samples collected during the preparation stage of the project in 2022 were determined by a Liquid Water Isotope Analyser (LWIA-24d, Los Gatos Research) at the Institute for Geological and Geochemical Research, Research Centre for Astronomy and Earth Sciences (IGGR). The overall uncertainty of these samples is ±1 ‰ for *δ*^2^H and ±0.2 ‰ for *δ*^18^O [17]. The deuterium excess (d−excess) was calculated as d−excess [‰] = *δ*^2^H − 8 x *δ*^18^O [18].

### 4.7 Measurement of the tritium content at ATOMKI

The determination of tritium (³H) in environmental water samples at low concentrations is commonly performed using the ³He ingrowth method, which relies on the radioactive decay of tritium to helium-3 (³He). Water samples are first degassed in a vacuum system to remove all dissolved gases, including any initial helium present in the sample. This step ensures that any subsequently measured ³He originates from in situ radioactive decay of ³H. After degassing, the samples are sealed and stored for a defined period, typically ranging from several weeks to months, during which tritium decays and produces measurable quantities of ³He (so-called tritiogenic ^3^He). The duration of this storage period directly influences the amount of ³He generated and thus the sensitivity of the measurement.

Following the ingrowth period, the accumulated gases are extracted from the sample and introduced into a purification system designed to isolate helium from other gas species. This purification is achieved using a combination of cryogenic trapping and adsorption techniques, where water vapor and condensable gases are removed at liquid nitrogen temperatures, and residual gases are separated using cryo-traps and adsorbents under controlled temperature conditions. The purified helium fraction is then introduced into a noble gas mass spectrometer for isotopic analysis.

Mass spectrometric measurement yields the abundances of both ³He and ⁴He. However, the measured ³He signal contains contributions from both tritiogenic ³He and background helium, which may originate from residual gases in the system or incomplete degassing. To isolate the tritiogenic component, the background contribution is estimated using the measured ⁴He abundance and the known atmospheric ³He/⁴He ratio. The tritiogenic ³He is then obtained by subtracting this background component from the total measured ³He. The ³H concentration of the original water sample is subsequently calculated from the amount of tritiogenic ³He, considering the decay constant of ³H, the elapsed times between sampling, extraction, and measurement, as well as the sample mass and any fractionation effects during sample preparation. To address this issue of the systematioc errors caused by air aliquots used during calibration, samples were spiked with ⁴He prior to measurement as described in [19].

### 4.8 Measurement of the tritium content of the samples at EUROFINS

The ^3^H measurements in the EUROFINS (former name: Wessling Hungary Ltd.) laboratory were performed using the analytical method based on the principle of selective isotopic enrichment using electrolysis. The volume of the water samples was reduced from 250 mL to 14 −15 mL by electrolytic enrichment, using systems with 15 cells with electronic control (Manufacturer: VITUKI, Budapest). The factor of ³H enrichment was about 15 to 16. The ³H activity of enriched water samples was counted by Quantulus 1220 Ultra Low-Level Liquid Scintillation Spectrometer (PerkinElmer), using SRM 4361C H-3 Radioactivity Standard (tritiated water, National Institute of Standards & Technology (NIST)). The detection limit was 0.5 TU = 0.06 Bq/L and the uncertainty ±5 - 10%.

### 4.9 Measurement of carbon isotope (CI) content of the samples at EUROFINS and ATOMKI

The determination of ¹⁴C in water samples is based on the analysis of dissolved inorganic carbon (DIC) using accelerator mass spectrometry (AMS). Groundwater samples are introduced into septum-sealed, gas-tight test tubes under a helium atmosphere [20,21]. Dissolved carbonate and bicarbonate species in the water are converted to CO₂ by the addition of phosphoric acid. The samples are subsequently heated (typically to approximately 75 °C) to enhance the transfer of CO₂ from the aqueous phase to the headspace. The degassing process follows Henry’s law, with increased temperature promoting the partitioning of CO₂ into the gas phase. Under the described conditions, a significant fraction of the dissolved carbon is released into the headspace of the sealed system.

The evolved CO₂ is transported from the reaction vessel using a controlled He carrier gas flow. During transfer, the gas stream is passed through phosphorus pentoxide to remove water vapor, and the CO₂ is trapped on a zeolite adsorbent. Following extraction, the CO₂ is either converted to graphite or measured directly in gaseous form. In the graphitization approach, CO₂ is reduced with hydrogen over a hot iron catalyst to produce solid graphite targets suitable for AMS measurement. Alternatively, the CO₂ can be introduced directly into the AMS using a gas ion source. In this configuration, a CO₂/He gas mixture is continuously delivered to the ion source at a controlled flow rate.

In both approaches, AMS is used to determine the isotopic ratio of ¹⁴C/¹^2^C. The instrument ionizes the carbon species, accelerates the ions, and separates them based on their mass-to-charge ratio, allowing the quantification of the ¹⁴C content. The results are reported as percent modern carbon (pMC), providing a measure of radiocarbon activity in the dissolved inorganic carbon fraction of the water sample.

### 4.10 Measurement of nitrate stable isotope (NSI) composition of the samples at JSI

Water samples were filtered within 12 h after sampling through 0.45 µm membrane filters and frozen at −24 °C until analysis. The dissolved nitrate was preconcentrated on ion exchange columns (BIO-RAD AG 1-X8, 200–400 mesh) and eluted with 1 M suprapure NaCl following the protocol of Silva et al. (2000) modified by Geng et al. (2015). The ^15^N/^14^N isotope analysis was performed after conversion of nitrate to N_2_O in aliquots containing 10 µg of nitrate-N following the denitrifier method (Sigman et al., 2001) using the Europa 20–20 isotope ratio mass spectrometer upgraded with a Sercon HS source assembly and 20–22 electronic suite (Sercon Ltd., Crewe, U.K.) coupled to the ANCA-TG trace gas separation module, where the thermodesorber was replaced with a home-made cryotrap. Results were reported as a relative *δ*^15^N value, representing a deviation of the ^15^N/^14^N ratio of the sample from that of the reference (air nitrogen), expressed in ‰. For calibration of the measurements to the Air scale, certified reference materials (USGS-34 and IAEA NO-3) were used. The USGS-35 reference material and an in-house working standard (KNO_3_ p.a., Merck) were used as controls. All reference materials and controls passed the same preparation procedure as water samples. The measurement uncertainty was determined as a long-term standard deviation of the *δ*^15^N measurements of working standards and was 0.5‰. All samples and reference materials were analysed in triplicate, and results were accepted if the standard deviations of the replicates in the same batch were within 0.2‰, which is the reproducibility declared by the IRMS producer. If the deviations were larger, measurements were repeated until they fell within acceptable limits.

### 4.11 Measurement of nitrate stable isotope (NSI) composition of the samples at IAEA

Nitrate (NO₃⁻) concentrations and stable isotope ratios (δ¹⁵N and δ¹⁸O) were measured from 20 mL of filtered groundwater samples, collected into clean HDPE sample bottles and preserved by adding 1 mL sulfanilic acid (2.5 mM in 10% HCl) in the field. Stable isotope ratios (δ¹⁵N and δ¹⁸O) were determined following chemical reduction of nitrate to nitrous oxide (N₂O) using titanium(III) chloride (TiCl₃). The analyses were conducted at the Czech Academy of Sciences (České Budějovice, Czech Republic). The analytical procedure followed the IAEA standard protocol (IAEA Nitrate SOP, Rev. 1.2, 2019), reducing nitrate to N₂O in a closed system, allowing accumulation of the produced gas in the vial headspace for subsequent isotopic analysis [22]. Due to the incomplete conversion efficiency of the Ti(III) reduction (∼65–75% yield), strict control of the sample-to-reagent ratio and consistent nitrate concentrations across samples, standards, and blanks was maintained to ensure reproducible isotope results. Quality assurance included routine analysis of blanks, standards, and duplicate samples.

## Limitations

Due to financial and technical constraints, not all parameters were determined for all collected samples. In particular, groundwater samples obtained from freshly dug wells frequently contained elevated levels of suspended material, which hindered filtration and precluded analysis of nitrate stable isotopes (δ¹⁵N and δ¹^8^O in NO₃⁻) and, in some cases, limited the determination of additional chemical parameters. This problem did not occur when sampling groundwater monitoring wells, especially deep ones.

Hydrological context data were not consistently available for all sampling events. Water level and discharge measurements were missing for certain locations and time periods, which may limit the direct linkage between concentration data and flow conditions.

For a small subset of privately dug wells, no detailed on-site sampling protocol was recorded. Consequently, key metadata such as groundwater levels at the time of sampling and total well depth are unavailable for these locations.

## Ethics Statement

The authors have read and follow the ethical requirements for publication in Data in Brief and confirm that the current work does not involve human subjects, animal experiments, or any data collected from social media platforms.

## Credit Author Statement

**Máté Krisztián, Kardos:** Conceptualization, Visualization, Writing - Original Draft, Data Curation; **Adrienne, Clement:** Conceptualization, Project administration, Funding acquisition; **Zsolt, Jolánkai:** Conceptualization, Formal analysis; **Ildikó, Musa:** Formal analysis, Investigation; **Radmila, Milačič Ščančar:** Investigation, Resources, Project administration, Funding acquisition; **Janez, Ščančar:** Investigation; **Miroslav, Štrbac:** Investigation; **Katarina, Kozlica:** Investigation; **Polona, Vreča:** Investigation, Methodology, Formal analysis, Data curation, Writing - Review & Editing; **Klara, Žagar:** Formal analysis; **Sonja, Lojen:** Investigation; **József, Deák:** Investigation, Methodology, Data curation, Writing - Review & Editing; **Palcsu, Laszlo:** Investigation; **Elemér, László:** Investigation; **István Gábor, Hatvani:** Conceptualization, Supervision, Writing - Review & Editing; **Zoltán, Kern:** Conceptualization, Supervision, Writing - Review & Editing.

## Data Availability

ARP Data RepositoryStable and radioactive isotope and multielement measurements from the Koppány and Ledava catchments (Original data).
